# Tissue-Cultured Chondrocytes Survive After Irradiation in 1300 Gy Dose

**DOI:** 10.3390/biomedicines13092153

**Published:** 2025-09-04

**Authors:** Denis Baranovskii, Anna Smirnova, Anna Yakimova, Anastas Kisel, Sergey Koryakin, Dmitrii Atiakshin, Michael Ignatyuk, Mikhail Potievskiy, Vyacheslav Saburov, Sergey Budnik, Yana Sulina, Vasiliy N. Stepanenko, Roman Churyukin, Bagavdin Akhmedov, Peter Shegay, Andrey D. Kaprin, Ilya Klabukov

**Affiliations:** 1 Department of Regenerative Medicine, National Medical Research Radiological Centre of the Ministry of Health of the Russian Federation, Koroleva st, 249036 Obninsk, Russia; 2 Scientific and Educational Resource Center for Innovative Technologies of Immunophenotyping, Digital Spatial Profiling and Ultrastructural Analysis, Patrice Lumumba Peoples’ Friendship University of Russia (RUDN University), 117198 Moscow, Russia; 3 Department of Biomedicine, University of Basel, Spitalstrasse 21, 4031 Basel, Switzerland; 4 Obninsk Institute for Nuclear Power Engineering, National Research Nuclear University MEPhI, 249020 Obninsk, Russia; 5 Center for Radiation Modification of Polymers “Teocortex Crosslink”, 249010 Borovsk, Russia; 6 Center for Synthetic Biotechnology, Sechenov University, 119991 Moscow, Russia; 7 Vishnevskiy National Medical Research Center of Surgery, 117997 Moscow, Russia

**Keywords:** cartilage, chondrocytes, cell death, extracellular matrix, ionizing radiation, radiobiology, radioresistance, scaffolds, tissue culture, tissue engineering

## Abstract

**Background/Objectives**: Radiobiology has shown heterogeneity in the sensitivity of cells to ionizing radiation, depending on a variety of conditions. The presence of an extracellular matrix (ECM) appears to confer a radioprotective effect on cells and can influence the cellular microenvironment by modulating the availability of oxygen and nutrients, which can affect cellular metabolism and stress responses. A three-dimensional cell culture allows the synergistic effect on cell survival to be obtained based not only on the radioprotective properties of the extracellular matrix but also on the stress-resistant endogenous properties of the cell culture. The aim of this study was to investigate the survival of chondrocytes in a 3D cell culture during high-dose ionizing irradiation. **Methods**: The properties of nasal chondrocytes were evaluated using a pellet culture model in which the cells were surrounded by a de novo synthesized extracellular matrix. Tissue cultures were exposed by gamma radiation at doses of 10, 100, and 1300 Gy. Cell viability was assessed after 2 days of irradiation by live/dead staining using confocal scanning laser microscopy. **Results**: Tissue-cultured chondrocytes survive after gamma-irradiation of low (10 Gy), medium (100 Gy), and high (1300 Gy) dosages; however, after irradiation of 1300 Gy, the percentage of surviving cells was lower. The average percentages of viable cells were evaluated as 82%, 79%, and 63% in low-, medium-, and high-dose groups, respectively. **Conclusions**: Under determined conditions, human cells are able to survive at doses of ionizing radiation that are significantly higher than the current limits.

## 1. Introduction

Recently, it has been observed that cell cultures exhibit significant mortality following exposure to ionizing radiation at doses ranging from 20 to 40 Gy [1,2,3,4]. However, these results were primarily obtained from experiments performed on cell culture monolayers. Interestingly, the presence of an extracellular matrix (ECM) appears to confer a protective effect on the cells, thereby enhancing their viability [5,6]. Despite this, most studies investigating cellular resistance to high doses of ionizing radiation are predominantly focused on therapeutic doses, often overlooking the potential implications of higher, non-therapeutic exposures.

The ability of living organisms and tissues to survive exposure to high doses of ionizing radiation is still not fully understood. Recent studies have investigated organisms with inherent radioresistant properties and the mechanisms underlying radioprotection development. Previous studies have shown that *Deinococcus radiodurans* can survive doses of γ-irradiation up to 2000 Gy due to endogenous mechanisms of archaeal radioresistance [7,8,9]. In relation to human cells, the potential pathways for inducing high-dosage radioprotection involve interactions between cells and symbiotic organisms [10], as well as the modification of ECM properties [11,12]. Chondrocytes are highly radioresistant because they have a low mitotic rate, a dense extracellular matrix, and efficient DNA repair mechanisms [13,14,15], and historical studies and in vitro work report that tumor chondrocytes survive even above 80 Gy [16]. Using 3D cell culture models instead of tissue explants has introduced a paradigm shift in our understanding of cellular responses to ionizing radiation. Unlike traditional 2D monolayers, 3D cultures more accurately mimic the in vivo cellular microenvironment, including cell–cell and cell–matrix interactions, nutrient gradients, and the presence of ECM [17]. These factors collectively contribute to a more physiologically relevant model for studying cellular behavior under stress conditions, such as exposure to ionizing radiation [18].

In 3D cell cultures, the ECM not only provides mechanical support but also plays a critical role in modulating cellular signaling pathways involved in survival, proliferation, and DNA repair mechanisms. This protective effect of the ECM may result in increased cellular resistance to ionizing radiation, potentially altering the threshold doses required to achieve similar levels of cell death observed in 2D cultures [19,20]. The 3D cultures may exhibit differential gene expression profiles and activation of survival pathways that are not present in 2D monolayers. For example, hypoxia-inducible factors and other stress response elements may be upregulated in 3D environments, contributing to increased radioresistance [21]. In addition, the spatial organization of cells in 3D cultures may influence the diffusion and potency of radiation-induced reactive oxygen species (ROS), further enhancing cell viability [22,23].

Previous studies have shown that triple freeze–thaw cycles are commonly used to create controlled cell death models in tissue samples and generate samples with predictable levels of cell death for experimental controls. These studies have also shown that triple freeze–thaw cycles are an effective method for inducing heat-shock stress [24,25,26]. The time-dependent period of cell observation depended on viability evaluations, including metabolic and proliferative activity, as well as delayed complications. Two days of irradiation is sufficient for developing measurable endpoints, such as DNA damage, oxidative stress, and apoptosis, for in vitro studies [27,28] and in vivo investigations [15,29,30]. Additionally, this period enabled us to avoid the secondary effects associated with prolonged cell growth or stress unrelated to radiation exposure evaluation. Mitotically dead cells undergo apoptosis as a secondary response, often referred to as delayed apoptosis, which is observed one to two days after irradiation [31,32,33,34].

We hypothesized that exposure of tissue cultures to high doses of ionizing radiation may lead to cell surveillance, especially cells with low metabolic activity like chondrocytes.

## 2. Materials and Methods

### 2.1. Study Design

**Figure 1:**
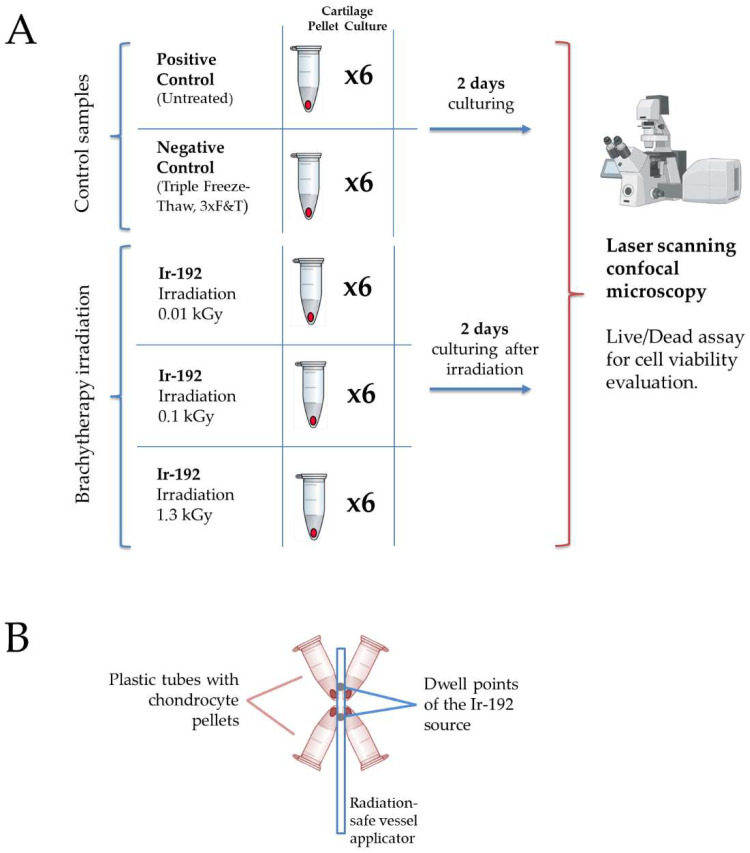
(**A**) The study design. Created with Biorender.com. (**B**) Schematic set-up for tube positioning around the Ir−192 brachytherapy source.

The study design of the radiobiological experiment is presented in Figure 1A. Schematic set-up for tube positioning is presented in Figure 1B.

### 2.2. Ionizing Irradiation

Gamma irradiation was performed on chondrocyte pellets derived from a patient at the University Hospital of Basel, with written informed consent and approval from the local ethics committee. Brachytherapy was carried out using gamma radiation from a sealed Iridium−192 (Ir−192) source. The Ir−192 source was calibrated and maintained according to institutional protocols to ensure consistent and accurate dosimetry.

The Ir−192 source, encapsulated in stainless steel and measuring 0.9 mm in diameter and 4.5 mm in length, was integrated into an afterloader system commonly used in brachytherapy. The Ir−192 source was stored in a radiation-shielded container and positioned at the treatment site using an applicator.

The administered dose during brachytherapy sessions was calculated based on a treatment plan tailored to the requirements of the experimental design. The irradiation doses were 10, 100, or 1300 Gy, depending on the experimental group. Chondrocyte pellets were mounted at the tips of Eppendorf plastic tubes (Eppendorf AG, Hamburg, Germany), with each sample positioned as close as possible to the optimized dwell positions of the Ir−192 source to minimize beam-on time. Sample locations were confirmed using computed tomography (SOMATOM Syngo CT, Siemens, Germany), which also served for dose calculations.

The small size of the pellet samples allowed for a relatively homogeneous dose distribution and efficient irradiation. Source positioning and dose optimization were performed using a commercial treatment-planning software (Oncentra Brachy, Version 4.5.2, Nucletron B.V., Netherlands). A schematic representation of the set-up, with four plastic tubes positioned around the applicator, is shown in Figure 1B.

### 2.3. Chondrocyte Sample Derivation

Nasal chondrocyte samples were used after obtaining written informed consent from the individuals and/or their relatives. The protocols of sample derivation were approved by the Local Ethical Commission (Switzerland; EKNZs; Ref.# 78/07) and the Local Ethics Committee of Sechenov University (Russia; Sechenov University; Ref.# 07–15).

### 2.4. Pellet Culture Chondrocytes

A pellet culture model of nasal chondrocytes was established according to a previously described protocol [35]. The pellets were used to evaluate the effect of different sources of ionizing radiation.

### 2.5. Cell Viability

Images, derived in the Biozentrum, University of Basel, were taken under a confocal laser scanning microscope Leica TCS SP8 (Leica Microsystems GmbH, Wetzlar, Germany), objective 20×, Z-stack step size 1 µm. Cell viability was assessed using confocal microscopy following the pre-staining of samples with calcein AM and ethidium homodimer−1, components of the Live/Dead Viability/Cytotoxicity Kit (Invitrogen, Carlsbad, CA, USA). Briefly, cells were cultured under the experimental conditions specified and then incubated with a staining solution containing 2 µM calcein AM and 4 µM ethidium homodimer−1 for 30 min at 37 °C in a humidified atmosphere with 5% CO_2_. Images were acquired from multiple random fields per sample to ensure representative sampling. Quantitative analysis of cell viability was performed using dedicated ImageJ (version 1.52a) software by counting live and dead cells.

### 2.6. Statistical Analysis

The data were obtained using six biological replicates per point. Data are presented as mean ± standard deviation (SD). Statistical analysis was performed using specific statistical software GraphPad PRISM v.8, applying one-way ANOVA with the significance level set at a *p*-value < 0.05.

## 3. Results

The pellet culture model of nasal chondrocytes shows significant differences from native cartilage, primarily characterized by a significantly higher and standardized number of cells per unit tissue volume. In this model, the newly formed tissue consists of chondrocytes and the cartilage ECM components synthesized by these cells.

Cultured pellets are compact, spherical aggregates of cells with a dense and uniform structure formed by the synthesized ECM. These pellets are typically rich in glycosaminoglycans and collagen, key components of the cartilage extracellular matrix, which contribute to their mechanical properties and functional similarities to native cartilage. The high cell density within these pellets facilitates robust matrix production, resulting in the formation of a tissue that, while not identical to native cartilage, closely mimics its biochemical and biomechanical properties.

**Figure 2:**
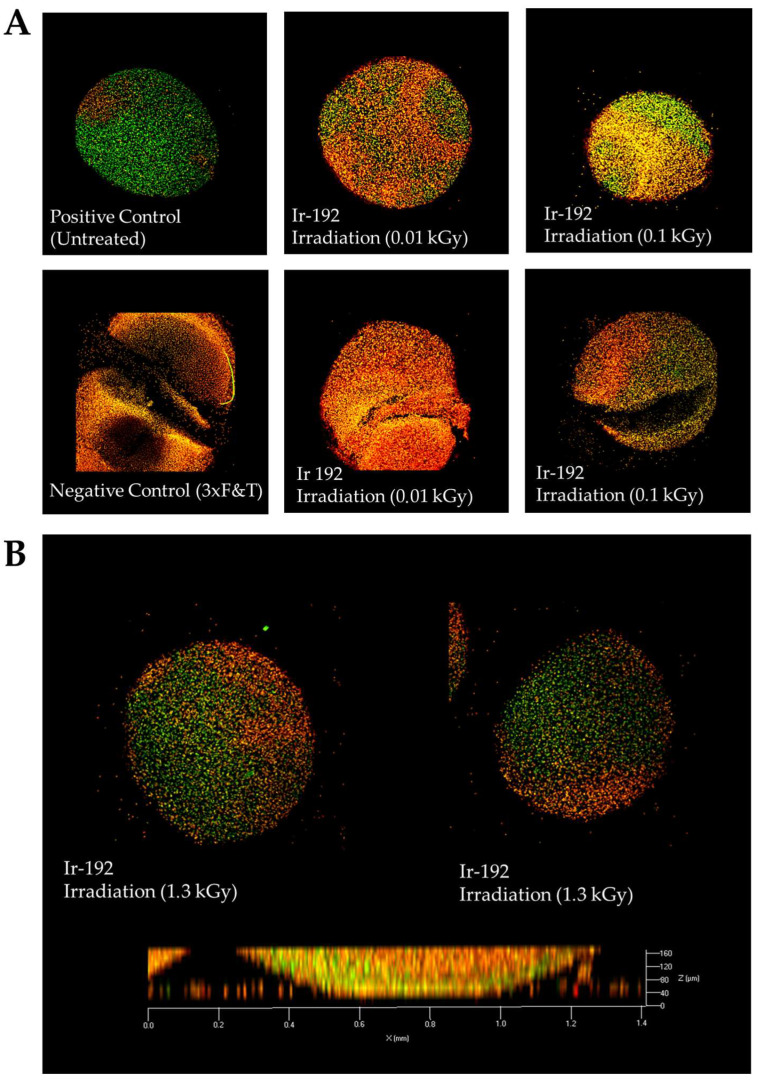
(**A**) Viability of nasal chondrocytes after exposure to different doses of gamma radiation; (**B**) Viability of nasal chondrocytes after exposure to gamma radiation at a dose of 1.3 kGy. Live/dead staining, confocal microscopy, 63× magnification. Dead cells are stained orange; live cells are stained green.

The irradiation of the pellet cultures with both low (Figure 2A) and high doses (Figure 2B) of gamma radiation was not responsible for all observed cell death, which can be explained by the low metabolic and proliferative activity of chondrocytes surrounded by de novo synthesized intercellular substances.

**Figure 3:**
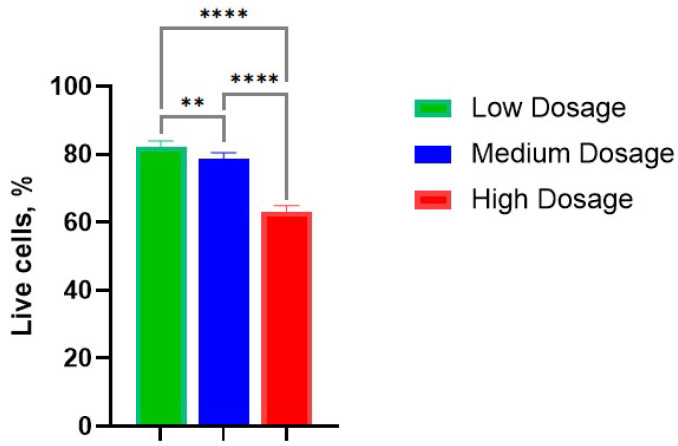
Survival rate of cells in the chondrocyte pellet culture in various dosages of gamma irradiation: Low—10 Gy; Medium—100 Gy; High—1300 Gy. **, *p*-value < 0.01; ****, *p*-value < 0.0001.

The average percentages of viable cells were evaluated as 82%, 79%, and 63% in low-, medium-, and high- dosage groups (Figure 3). It is not surprising that the percentage of living cells decreased with dosage rate; however, some cells remained alive and retained cellular respiration.

A similar effect was observed during the electron beam irradiation of the chondrocyte pellet culture in the same conditions (data presented in Supplementary Files S1 and S2).

## 4. Discussion

The interaction of cells with the ECM modulates many important processes such as proliferation, differentiation, migration, and survival [36]. The ECM could assist cell viability and improve cell resistance to ionizing radiation, chemicals, hypoxia, and other external factors, but these effects seemed to be minor. Extreme cases of cell viability were observed in some animals, but there were no translations to humans or vertebrate animals [37,38].

The nature of induced cell radioresistance following residence in the ECM is multifaceted, influenced by several key factors. The stiffness and elasticity of the ECM can alter cellular behavior and enhance a cell’s ability to withstand radiation damage [39]. The reorganization and stabilization of the cytoskeleton in response to ECM mechanical signals can enhance cellular resilience by improving structural integrity and facilitating efficient repair mechanisms [40]. The biochemical composition of the ECM, including the presence of growth factors, cytokines, and other signaling molecules, can activate survival pathways and enhance DNA repair processes. The interaction between cell surface receptors and ECM components can trigger intracellular signaling cascades that promote cell survival and reduce apoptosis in response to radiation [41]. Additionally, the ECM can influence gene expression profiles, leading to the upregulation of genes associated with DNA repair, antioxidant defense, and anti-apoptotic pathways [42]. Chondrocyte regulation differs between cellular subpopulations and can affect viability and survival through intrinsic genomic and epigenetic mechanisms [43].

Moreover, the ECM can impact the cellular microenvironment by modulating the availability of oxygen and nutrients, which can affect cellular metabolism and stress responses. Hypoxic conditions within the ECM, for instance, can activate hypoxia-inducible factors (HIFs) that promote cell survival under stress conditions, including radiation exposure [44].

The mechanisms of cell death during exposure to ionizing irradiation have been widely investigated; however, cell death, viability, and anastasis in tissue-engineered constructs after ionizing radiation exposure have not been examined in detail [45,46,47], and the range of resistance of cells of different tissues to ionizing radiation and the specific mechanisms of cell death remain insufficiently studied. Therefore, the heterogeneity of the types of cell death [48], coupled with the phenomenon of cellular anastasis, can obscure the extent of damage following irradiation and highlight the intracellular mechanisms of enhanced radioresistance, as well as highlighting the need for studies aimed at reducing these mechanisms for the stimulation of radiosensibilization in tumor cells [11,49]. Last but not least is the fact that chondrocytes which are highly resistant to irradiation could be used in long-term space exploration [50]. There is no doubt that these findings could be used to investigate ways to increase the radioresistance of human and animal cells for long-term space exploration. In addition, the differences between ECM properties in tumor and normal tissues could be used in advanced protocols for radiotherapy with ECM-targeted adjuvants [51].

A notable limitation of the present study was the lack of a comprehensive assessment of cell proliferative activity. This could have been achieved by using Ki−67 expression immunohistochemistry assays, which are widely recognized for their ability to indicate active cell proliferation. In addition, evaluation of the colony-forming capacity of the cells would have provided direct insight into their proliferative potential. The use of chondrocyte pellets allowed us to obtain the synergetic effect on cell survival based not only on the radioprotective properties of the ECM but also the stress-resistance endogenic properties of the chondrocyte cell culture.

## 5. Conclusions

Tissue-cultured chondrocytes are capable of survival after irradiation at doses of at least 1300 Gy. This remarkable radioresistance demonstrates the resilience of these specialized cells and suggests that certain differentiated cell types may have unique structural or metabolic adaptations that confer exceptional tolerance to DNA damage and oxidative stress. This observed effect could be applied to space exploration and to developing advanced techniques for chondrosarcoma radiotherapy.

## Data Availability

The data presented in this study are available on request from the corresponding author.

## Supplementary Materials

The following supporting information can be downloaded at: https://www.mdpi.com/article/10.3390/biomedicines13092153/s1, File S1: Electron beam irradiation experiments; File S2: LAS raw data file.

## Abbreviations

The following abbreviations are used in this manuscript:

DNA	Deoxyribonucleic acid
ECM	Extracellular matrix
