# Supplementary material for: Tissue-Cultured Chondrocytes Survive After Irradiation in 1300 Gy Dose
Source: Biomedicines. 2025 Sep 4;13(9):2153. doi: 10.3390/biomedicines13092153 (PMC12467947; doi:10.3390/biomedicines13092153)
Supplement: Supplementary file 1 [file biomedicines-13-02153-s001.zip › Supplement 1.pdf]

Supplement 1

Electron beam irradiation study was performed for evaluation of viable cells in pellets (Figure A1). Chondrocyte pellets were based on cells obtained from a patient at the Sechenov University, with written informed consent and approval from the Local ethics committee of the Sechenov University. Electron irradiation was performed on the Tekhleor Electron-beam Irradiation facility (Tekhleor LLC, Vorsino, Russia) in dosages 80 Gy and 1200 Gy. We used the Novac-11 standard built-in equipment and software to control the electron beam irradiation dose. Images were taken under Leica DMI4000 laser scanning confocal microscope (Leica Microsystems GmbH, Wetzlar, Germany).

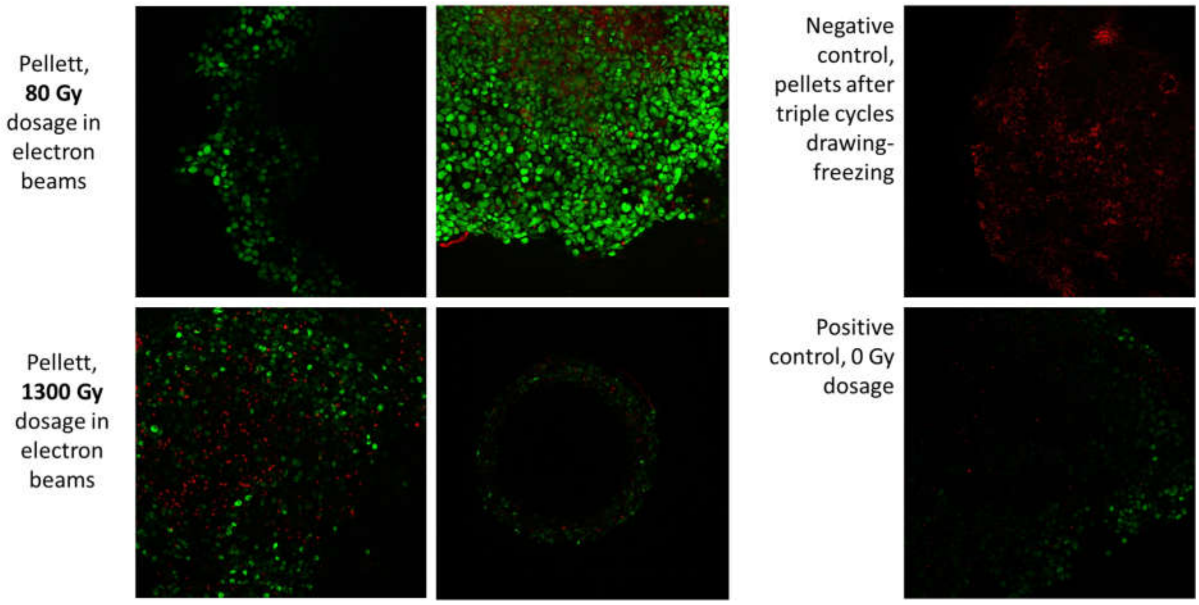

Figure S1: Evaluation of viability of chondrocytes in pellets after irradiation of electron beams in varying dosages. LIVE-DEAD staining, confocal microscopy. Dead cells are stained orange, live cells are stained green. Confocal laser microscopy
